# Beyond Overuse: A Cross-Sectional Analysis of Insulin Resistance in De Quervain’s and Stenosing Flexor Tenosynovitis

**DOI:** 10.5704/MOJ.2603.010

**Published:** 2026-03

**Authors:** EM de-la-Paz

**Affiliations:** Department of Orthopaedics, The Medical City Clark, Angeles, Philippines

**Keywords:** insulin resistance, metabolic syndrome, de Quervain’s tenosynovitis, stenosing flexor tenosynovitis, cross-sectional analysis

## Abstract

**Introduction:**

Insulin resistance (IR) or metabolic syndrome (MetS) may escalate the propensity for tenosynovitis from accrual of advanced glycation end products from chronic hyperglycaemia. This study characterised IR/MetS among patients with de Quervain’s tenosynovitis (DQT) and trigger finger (TF) or stenosing flexor tenosynovitis in a clinical practice setting.

**Materials and methods:**

A prospective, non-interventional, cross-sectional study enrolled 118 patients with DQT or TF at a general orthopaedics-hand surgery practice. IR/MetS was defined using established criteria, including HbA1c levels, a prior diagnosis of prediabetes (PD) or type 2 diabetes (T2D), and Japanese diagnostic guidelines. The proportion of IR was analysed using descriptive statistics. Patient characteristics and metabolic parameters were summarised and compared between IR and non-IR groups.

**Results:**

Of 93 patients included in the final analysis, 65 (70%) were insulin resistant. Among IR patients, 47% had PD, and 23% had T2D. No significant gender differences were observed. Patients with IR had higher mean BMI, blood uric acid, fasting blood sugar, HbA1c, and TG:HDL-C ratios compared to non-IR patients. The distribution of TF and DQT was similar between groups.

**Conclusion:**

This study describes the metabolic profile of patients with tenosynovitis, revealing a high proportion of IR/MetS. These findings highlight metabolic dysfunction as a potential factor in chronic tendinopathies and underscore the need for further research into underlying mechanisms and targeted interventions.

## Introduction

Insulin Resistance Syndrome (IR), formerly known as Syndrome X and now also acknowledged as Metabolic Syndrome (MetS), has been extensively studied for its associations with various diseases since its recognition^1^. It is the most prevalent health disorder worldwide^2^, increasing the risk of numerous chronic disorders such as prediabetes (PD), type 2 diabetes mellitus (T2D), obesity, metabolic-associated fatty liver disease, atherosclerotic cardiovascular disease, neurodegenerative conditions, infertility, certain cancers and even various musculoskeletal conditions^3^.

The link between diabetes and hand pathologies has long been established^4^. However, the management of conditions such as de Quervain’s tenosynovitis (DQT) and trigger finger (TF) remains largely symptom-centric, relying on steroid injections or tendon sheath incisions to provide temporary relief^5^. This approach fails to address potential underlying metabolic contributors, often resulting in suboptimal outcomes and high recurrence rates. Despite the well-known systemic effects of IR and MetS, their role in the pathogenesis of tenosynovitis remains under-explored, leaving a critical gap in our understanding of these conditions.

This study investigates the proportion of IR/MetS among patients with DQT and TF, aiming to better characterise the metabolic profile of affected individuals. By identifying patterns of metabolic dysfunction in tenosynovitis patients, this investigation seeks to provide a foundation for future investigations into the role of IR/MetS in chronic tendinopathies and the potential for mechanism-based management strategies.

## Materials and Methods

This was a prospective, observational, cross-sectional study conducted in a clinical practice setting at a solo general orthopaedics-hand surgery practice affiliated with three private hospitals from February 2023 to January 2024. No IRB approval was procured for this non-interventional study that does not reveal any private patient information. The study adhered to the ethical standards for patient treatment outlined in the Helsinki Declaration of 1975, as revised in 1983.

Eligible participants included men and women with age ≥21 years diagnosed with DQT or TF. Patients without a recent serum metabolic workup were excluded from the study.

All data were collected during consultation and stored in the electronic medical record, OpenEMR 7.0 (open-emr.org). Pertinent histories of both hand pain and metabolic health were obtained. Body weight, height, and blood pressure were recorded, and body mass index (BMI) was calculated (weight in kilograms divided by height in meters squared, kg/m^2^). The diagnosis of DQT was confirmed based on tenderness over the first dorsal compartment and a provocative Finkelstein manoeuvre, while the diagnosis of TF was confirmed by eliciting tenderness over the A1 pulley on the palm, with or without catching or locking of the involved digit.

Patients were classified as IR if they met at least one of three criteria (Table I): the Japanese Criteria for Metabolic Syndrome^6^, an HbA1c level of ≥5.7%^7^, or a preexisting diagnosis of PD or type 2 diabetes with medication use. Preexisting diagnoses of PD or T2D were made by the patients’ primary care physician or diabetologist/endocrinologist before presentation for the hand-related concerns. In cases where patients were identified as IR but had no PD or T2D diagnosis, classification was based on HbA1c values, with levels of 5.76.4% indicating PD and levels ≥6.5% indicating T2D^7^.

**Table I:** Criteria used for diagnosing insulin resistance/metabolic syndrome

Criteria	Parameters
A. Japanese criteria, 2017^6^	BMI ≥25, plus any three of the following: 1. Elevated Waist Circumference (Men: ≥85cm, Women: ≥90cm) 2. Elevated BP (SBP ≥130mmHg, or DBP ≥85mmHg, or on Rx) 3. Elevated FBS (≥100mg/dL) or HbA1c ≥5.6% or on maintenance medications 4. Elevated TG (≥150mg/dL) 5. Reduced HDL-C (≤40mg/dL)
B. Glycosylated hemoglobin^7^	HbA1c ≥5.7%
C. Pre-existing prediabetes or type 2 diabetes with medication use (diagnosed by primary care physician, endocrinologist or diabetologist)	Current medications of participants: 1. Biguanides (metformin) 2. Sulfonylureas (gliclazide, glibenclamide, glipizide) 3. DPP-4 inhibitors (linagliptin) 4. SGLT2 inhibitors (empagliflozin) 5. GLP-1 agonists (semaglutide) 6. Others: LDL-lowering drugs (atorvastatin, rosuvastatin), various anti-hypertensive drugs, blood thinners (clopidogrel, aspirin), anti- hyperuricemia drugs (allopurinol, febuxostat), fatty acid oxidation inhibitors (trimetazidine)

A total of 118 consecutive patients met the inclusion criteria during the study period. All patients, except those who had the tests within the past three months, were verbally consented to undergo a routine serum metabolic workup consisting of fasting blood glucose (FBS), triglycerides (TG), high-density lipoprotein cholesterol (HDL-C), low-density lipoprotein cholesterol (LDL-C), blood uric acid (BUA) and glycosylated haemoglobin (HbA1c). Twenty-five patients declined metabolic testing and were excluded. Patients who had a metabolic workup within the prior three months were not required to repeat testing. Among the included patients, 26 with pre-existing PD and all 21 with T2D with medication use automatically fulfilled the IR criteria.

All patients were offered the standard treatment options for their hand conditions, including activity modification, non-steroidal anti-inflammatory drugs, cortisone injection, and tendon sheath incision when indicated. The shared decision on the choice of treatment was not influenced by this investigation. No pharmacologic interventions targeting metabolic comorbidities were prescribed or modified during the study. Instead, all patients received counselling on the potential benefits of therapeutic carbohydrate restriction for long-term management of both tendinopathy and IR/MetS.

The primary outcome was the proportion of IR in this cohort of tenosynovitis patients. Descriptive statistics were used to summarise patient characteristics. Categorical variables, including IR status, gender, and the presence of TF or DQT, were reported as frequencies and percentages. Continuous variables, including BMI, blood uric acid, fasting blood sugar, HbA1c, LDL-C, and TG:HDL-C ratio, were reported as means with standard deviations for normally distributed data or medians with interquartile ranges for non-normally distributed data. The normality of continuous variables was assessed using the Shapiro-Wilk test, with p<0.05 indicating non-normality. Normally distributed variables were compared using independent samples t-tests, with 95% confidence intervals reported, while non-normally distributed variables were compared using the Wilcoxon rank-sum test with continuity correction, with medians and interquartile ranges reported.

Statistical analyses were performed using R 4.2.3 (R Foundation for Statistical Computing, www.r-project.org). R codes for statistical analyses were generated with the assistance of OpenAI’s ChatGPT-4. A significance level of p<0.05 was used for all statistical comparisons.

## Results

Among the 93 patients with tenosynovitis, 65 (70%) were IR, while 28 (30%) were non-IR (Table II). Of the 93 participants, 44 (47%) had PD, and 21 (23%) had T2D, leaving only 28 (30%) classified as metabolically healthy. Notably, 18 (19%) had undiagnosed PD prior to seeking consultation for their hand problems.

Patients with IR tended to be older, with a mean age of 51 years compared to 44 years in the non-IR group. Significant differences were also observed in physical and serum metabolic markers. The IR group had higher mean values for BMI (29 vs 25), blood uric acid (BUA; 8.1mg/dL vs 6.2mg/dL), fasting blood sugar (FBS; 128mg/dL vs. 96mg/dL), glycosylated haemoglobin (HbA1c; 6.2% vs 5.4%), and triglyceride-to-HDL cholesterol ratio (TG:HDL-C; 3.4 vs 1.9). While low-density lipoprotein cholesterol (LDL-C) tended to be lower in the non-IR group (128mg/dL vs 136mg/dL), this difference was not statistically significant.

**Table II:** Comparison of metabolic and demographic characteristics between insulin resistant and non-insulin resistant patients.

Characteristic	Insulin Resistant	Non-Insulin Resistant	Difference 95% CI	P-value
Total, N=93	65 (70%)	28 (30%)		
Type 2 Diabetes	21 (23%)			
Prediabetes	44 (47%)			
Female	44 (68%)	17 (61%)		
Male	21 (32%)	11 (39%)		
Hand Involvement	TF: 41 (63%)	TF: 19 (68%)		
	DQT: 20 (31%)	DQT: 9 (32%)		
	Both: 4 (6%)	Both: 0 (0%)		
Age, years, ( x ± SD)	51 ± 12	44 ± 11	[2.04, 12.29]	0.01
BMI, kg/m^2^, ( x ± SD)	29 ± 3.2	25 ± 1.8	[2.87, 4.97]	<0.001
BUA, mg/dl, ( x ± SD)	8.1 ± 1.9	6.2 ± 1.4	[1.19, 2.62]	<0.001
FBS, mg/dL, ( x ± SD)	128 ± 33	96 ± 7	[23.07, 40.36]	<0.001
LDL-C, mg/dL, ( x ± SD)	136 ± 33	128 ± 31	[-6.89, 21.82]	0.30
HbA1c, %, x̃ (IQR)	6.2 (IQR 5.9-7.2)	5.4 (IQR 5.1-5.5)		<0.001
TG:HDL-C Ratio, x̃ (IQR)	3.4 (IQR 2.4-3.9)	1.9 (IQR 1.6-2.1)		<0.001

Abbreviations – BMI = body mass index, BUA = blood uric acid, FBS = fasting blood sugar, LDLC = low density lipoprotein cholesterol, HbA1c = glycosylated hemoglobin, TG:HDL-C = triglyceride to high density lipoprotein cholesterol ratio

Among the 65 IR patients, 41 (63%) had TF, and 20 (31%) had DQT (Table II). In contrast, among the 28 non-IR patients, 19 (68%) had TF, and 9 (32%) had DQT. The proportion of patients with TF and DQT was similar between IR and non-IR groups. Additionally, 4 (6%) of the T2D patients, whereas none in the PD group, had both TF and DQT.

A notable subset of six IR patients developed DQT postpartum, with symptoms emerging within two months of childbirth. Additionally, seven IR patients (six with T2D and one with PD) had a history of previously treated TF but presented with new tenosynovitis symptoms. While not a primary focus of this study, these patterns may warrant further investigation.

## Discussion

This non-interventional, cross-sectional study describes the metabolic characteristics of patients with tenosynovitis and identifies a high proportion of IR within this cohort. The findings suggest that metabolic dysfunction may be more common among individuals with tenosynovitis, though the study design does not allow for causal inferences. These results align with previous studies showing that individuals with T2D have an increased occurrence of hand abnormalities^8^, T2D is an important risk factor specifically for TF^9^, and that there is a correlation between rising HbA1c levels and the development of TF^10^. While this study does not establish a definitive link between IR and tenosynovitis, the observations contribute to the growing body of research exploring potential metabolic influences on musculoskeletal conditions.

In this study, the majority of tendinopathies manifested before a formal diagnosis of T2D. Among the 93 participants, 47% had PD, and 23% had T2D, with 19% being unaware of their hyperglycaemic state before seeking consultation for their hand problems. These findings suggest that orthopaedic surgeons may have an opportunity to recognise metabolic dysfunction in patients presenting with tendinopathies, potentially facilitating earlier referral and intervention.

The IR group exhibited higher mean BMI, reflecting the increased likelihood of hyperglycaemia in overweight or obese individuals. Chronic hyperglycaemia, as indicated by elevated HbA1c levels in IR participants, may lead to the formation of advanced glycation end products (AGEs) produced by non-enzymatic (Maillard) reaction between glucose and proteins^11^. AGEs create pathological cross-links in tendon collagen making them thicker, less flexible and more resistant to movement^12^. Additionally, AGEs disrupt normal inflammatory mediator function by binding to receptor for AGEs (RAGE), increasing fibrosis and contributing to the symptoms observed in DQT and TF^11^. These mechanisms support a strong association and suggest a potential causal link, though causality cannot be confirmed due to the observational design of this study.

Other metabolic markers, such as TG:HDL-C ratio, LDL-C, and BUA, though not necessarily contributing to the pathogenesis, were also associated with tendinopathies. The TG:HDL-C ratio, in particular, has been proposed as a simple indicator of IR^13^, with higher values reflecting poorer metabolic health. Except for LDL-C, targeted for lowering by statins that some of the IR patients were on, all serum markers were associated with tenosynovitis despite participant heterogeneity. While these markers may not directly contribute to tenosynovitis, their association with IR suggests a broader systemic metabolic dysfunction among affected patients.

Certain observations in this study raise additional clinical questions. Six IR patients developed DQT postpartum, with symptoms emerging within two months of childbirth. These patients had no prior history of IR or wrist pain, highlighting the physiological IR that develops during pregnancy. Additionally, important to note that the pathogenesis of tenosynovitis in these patients can occur within the few months of gravidity. Seven patients with IR (6 T2D, 1 PD) had a history of resolved TF but presented with new tendinopathies after several months or years. These findings illustrate the limitations of current treatment paradigms, which often fail to address possible underlying metabolic causes of tendinopathies. While these patterns were not the primary focus of this study, they warrant further exploration in future research.

The conventional understanding of DQT and TF emphasises mechanical overuse as the primary cause, leading to treatments focused on symptom relief, such as steroid injections or tendon sheath incisions^5^. However, the high proportion of IR in this cohort suggests that metabolic dysfunction may play a contributory role. Furthermore, the absence of multiple tendon pathologies in the PD group, compared to their presence in a subset of T2D patients, raises the possibility that more advanced hyperglycaemia predisposes individuals to multiple tendon disorders. These findings highlight the potential value of screening for IR in patients with chronic tendinopathies to better understand underlying systemic contributors. While pharmacologic interventions for IR may be outside the scope of orthopaedic practice, lifestyle modifications, including dietary changes and increased physical activity, have shown promise in improving insulin sensitivity and overall metabolic health^14,15^. Emerging evidence supports the role of therapeutic carbohydrate restriction and nutritional ketosis in reversing IR and improving glycaemic control ^16,17^, which could potentially influence the course of chronic musculoskeletal conditions. In osteoarthritis for instance, the pain relief observed with as little as 5% weight loss cannot be attributed solely to the resulting off-loading of the diseased joint^18^, but perhaps also due in part to improving insulin sensitivity and enhancement of cardio-metabolic profile.

Several limitations must be acknowledged. The findings may not be generalisable beyond the specific setting of a solo general orthopaedic practice. Patient demographics and healthcare access factors may differ between clinical settings, potentially influencing the observed proportion of IR. The inclusion of two distinct conditions, DQT and TF, assessed together for the numbers, may introduce heterogeneity, though their underlying pathophysiology is similar. Selection bias cannot be ruled out, as 21% of eligible patients were excluded due to a lack of metabolic workup, which may have been influenced by factors such as healthcare access and insurance status. Recall bias may also affect the accuracy of patient-reported diagnoses of PD and T2D. The duration of hyperglycaemia, a key factor in tendinopathy development, was not assessed. While the homeostatic model assessment for insulin resistance (HOMA-IR) is considered the standard for evaluating IR^19^, this study relied on alternative validated measures due to practical constraints. The unavailability of fasting insulin testing in many localities limits the use of HOMA-IR, and even among endocrinologists and diabetologists, familiarity with this parameter may vary. Albeit, values of HbA1c ≥5.7% have also been shown to reliably correlate with IR^7^. Finally, the heterogeneity of medications for comorbid metabolic conditions may have influenced the results of certain metabolic markers like LDL-C.

While this study provides valuable insights into the metabolic characteristics of patients with tenosynovitis, it does not confirm causality. Future studies should employ longitudinal cohort designs to assess IR at baseline and track the incidence of tenosynovitis over time. The experiences of postpartum patients who developed DQT within a few months of pregnancy suggest that studying the temporal relationship between IR and tendinopathy may be very feasible. A prospective case-control study could further clarify the metabolic differences between individuals with and without tenosynovitis. Other conditions such as early onset osteoarthritis^20^, osteoporosis^21^, lumbar spinal stenosis^22^, crystalline arthropathy^23^, and femoral head avascular necrosis^24^ may also be associated with IR or MetS and should be put under a clinical lens by the orthopaedic community. Exploring the temporal correlation between improving insulin sensitivity and the resolution of chronic musculoskeletal conditions represents another promising avenue for investigation.

## Conclusion

IR/MetS is commonly observed in patients with DQT and TF. A substantial proportion (70%) of IR/MetS among tenosynovitis patients underscores the potential role of metabolic dysfunction as a contributing factor in chronic tendinopathies. These findings emphasise the need for future research to further explore the relationship between IR/MetS and chronic orthopaedic conditions, including whether metabolic interventions could influence disease progression. A greater understanding of these associations may help shift management strategies beyond symptom-focused treatments toward addressing underlying metabolic factors.
